# Visceral adiposity indices and cardiometabolic risk markers in patients with hypertension

**DOI:** 10.20945/2359-3997000000536

**Published:** 2022-12-01

**Authors:** Berilany dos Santos Sena, Fabiana Cristina Lima da Silva Pastich Gonçalves, Regiane Maio, Rebecca Peixoto Paes Silva, Maria da Conceição Chaves de Lemos, Ilma Kruze Grande de Arruda

**Affiliations:** 1 Universidade Federal de Pernambuco Recife PE Brasil Programa de Pós-graduação em Nutrição, Universidade Federal de Pernambuco, Recife, PE, Brasil; 2 Universidade Federal de Pernambuco Departamento de Nutrição Recife PE Brasil Departamento de Nutrição, Universidade Federal de Pernambuco, Recife, PE, Brasil

**Keywords:** Adiposity, cardiovascular diseases, hypertension, nutritional status

## Abstract

**Objective:**

Arterial hypertension (AH) is a risk factor for cardiovascular diseases (CVD). We sought to evaluate the association between two adiposity indices (visceral adiposity index [VAI] and lipid accumulation product [LAP]) with traditional markers of cardiometabolic risk in hypertensive patients.

**Materials and methods:**

This is a cross-sectional study with 1,273 subjects with hypertension treated as outpatients at a university hospital. The VAI and LAP were calculated using formulas stratified by sex. Cardiometabolic risk variables were considered: overweight, risk for waist circumference (WC), waist-to-hip ratio (WHR), waist-to-height ratio (WHA), and altered biochemical test values. The predictive effect of independent variables on outcomes was assessed by multivariate linear regression analysis. There was statistical significance when p ≤ 0.05.

**Results:**

Higher cardiometabolic risk (according to BMI, WHR, WHA, and altered biochemical parameters) was associated with higher values of VAI and LAP with statistical significance (p ≤ 0.05). The regression models used explained 30.7% and 10.5% of the changes in LAP and VAI, respectively.

**Conclusion:**

LAP and VAI are associated with cardiometabolic risk parameters in the individuals evaluated, suggesting that these indices can be used to screen for CVD risk in individuals with AH.

## INTRODUCTION

Arterial hypertension (AH) is a multifactorial chronic non-communicable disease (NCD) characterized by persistent elevation in blood pressure levels: systolic blood pressure ≥ 140 and/or diastolic blood pressure ≥ 90 mmHg ( 1 ). According to the World Health Organization (WHO) ( 2 ) in 2017, approximately 1.13 billion of individuals in the world were hypertensive. Based on the national prevalence ( 3 ), self-reported hypertension increased from 22.6% in 2006 to 24.5% in 2019, being higher in women and older individuals.

It is known that AH is the main risk factor for cardiovascular diseases (CVD). It holds a significant contribution to the increase in morbidity and mortality ( 4 ). Therefore, it is important to adopt simple, practical tracing parameters with a predictive potential. Anthropometric variables, although presenting limitations, are risk predictors for cardiometabolic disorders ( 5 ), while changes in laboratory markers indicate a risk factor for atherogenesis ( 6 ). In this context, clinical indicators that combine anthropometric and biochemical measurements may represent the interaction between excess body visceral adiposity and cardiometabolic risk ( 7 ).

In this sense, some parameters have been proposed to estimate the amount of visceral adipose tissue related to adverse outcomes, such as the lipid accumulation product (LAP), derived from the product between waist circumference (WC) and fasting triglyceride concentration (TG), and visceral adiposity index (VAI), an empirical mathematical model based on the association of anthropometric measurements (WC and body mass index (BMI)) with laboratory parameters (TG and high-density lipoprotein cholesterol [HDL-c]) ( 7 ).

Previous studies have argued for LAP ( 8 ) and VAI ( 9 ) as indices with a potential to predict lipid accumulation and visceral fat function associated with adverse cardiovascular and metabolic outcomes, as well as significant association of LAP with increased risk of AH ( 10 ) and a positive relationship between VAI levels and proteinuria in hypertensive individuals ( 11 ). However, anthropometric and biochemical markers of cardiometabolic risk were not associated with LAP and VAI among the Brazilian population with hypertension. Thus, the present study aims to evaluate the association between two visceral adiposity indices and anthropometric and biochemical markers of cardiometabolic risk in hypertensive patients.

## MATERIALS AND METHODS

### Study design, period, and location

This is a cross-sectional study conducting the analysis of a research database. The study is entitled ‘Kidney disease in hypertensive patients with metabolic syndrome’ and was carried out with patients treated at the Hypertension Clinic of the Cardiology Service of the *Hospital das Clínicas* of the Federal University of Pernambuco (HC/UFPE) from January 1996 to July 2011. Data collection was carried out in four periods. In this study, the collected and transcribed data referring to the baseline were used.

### Study population and eligibility criteria

The population consisted of patients with AH, adults and elderly, of both sexes, followed up at the clinic at the first moment of collection.

To eliminate possible biases, we excluded from the baseline survey patients with secondary AH (n = 5), cardiac arrhythmias/electrical conduction disturbances on the electrocardiogram (n = 26), heart failure (n = 7), previous myocardial infarction (n = 9), dyslipidemia with the use of lipid-lowering medication (n = 46), and *diabetes mellitus* (n = 203), and patients with incomplete data in the medical records (n = 553). The final sample of the present study included 1,273 individuals.

### Visceral adiposity indices

The VAI was calculated by the equation proposed by Amato and cols. ( 12 ) stratified according to sex, where WC is expressed in cm, BMI in kg/m², and TG and HDL-c values in mmol/L. For men: VAI = [WC / 39.68 + (1.88 × BMI)] × (TG / 1.03) × (1.31 / HDL-c); for women: [WC / 36.58 + (1.89 × BMI)] × (TG / 0.81) × (1.52 / HDL-c).

The LAP was obtained using a specific formula for each sex. For women: (WC [cm]-58) × (TG [mmol/L]); for men: (WC [cm]-65) × (TG [mmol/L]) ( 13 ).

Due to the lack of consensus regarding cut-off points for both the LAP and the IAV that would allow their categorization into normal and high values, they were analyzed continuously.

### Cardiometabolic risk markers

The following cardiometabolic risk variables were considered: overweight assessed by BMI, risk by WC, waist-to-hip ratio (WHR) and waist-to-height ratio (WHA), serum levels of low-density lipoprotein cholesterol (LDL-c), total cholesterol (TC), high fasting and postprandial glycemia, and glycated hemoglobin (HbA1c) and low HDL-c.

### Anthropometric variables

Weight and height were measured using standardized techniques ( 14 ). BMI was obtained by the ratio between weight and squared height. The cutoff points recommended by the WHO ( 15 ) for adults and Lipschitz ( 16 ) for the elderly were considered for BMI classification. The data obtained were categorized into malnutrition/normal weight and overweight, the latter including overweight and obesity in adults.

WC was measured and classified according to WHO criteria ( 17 ) considering the cutoff points ≥ 80 cm for women and ≥ 94 cm for men as a risk for CVD. The hip circumference (HC) was measured at the pubic symphysis with a tape measure encircling the hip in the region of greatest protuberance of the glutes.

The waist-to-hip ratio (WHR) was obtained from the ratio between WC and HC and the cutoff points ≥ 0.90 for men and ≥ 0.85 for women were used as a risk indicator ( 18 ).

Furthermore, the waist-to-height ratio (WHA) was obtained by dividing WC by height, adopting the cutoff point ≥ 0.5 for cardiometabolic risk ( 19 ).

### Biochemical variables

Blood collection was performed after fasting for 10 to 12 hours, at the Laboratory Unit of the HC/UFPE (ULAB-HC-UFPE). For biochemical tests, the Analytical Standard Operating Procedure of the Biochemistry Sector of the Laboratory Unit (ULAB) of the hospital was followed, which uses the automated Dimension AR-Dade Behring equipment and a Centrifuge to separate serum and red blood cells. The cut-off points of the Brazilian societies of diabetes ( 20 ) and cardiology ( 21 ) were used to evaluate the fasting glucose, postprandial glucose, HbA1C and total cholesterol, LDL-c, HDL-c, TG respectively.

### Demographic and lifestyle variables

Age (complete years), sex (female or male), and self-reported color (white and non-white) were analyzed. The practice of regular physical exercise reported and guided by a professional was considered when there was practice at least three times a week, for about 60 minutes. In terms of smoking, the habit of smoking at least one cigarette per day was considered, and individuals were stratified as smokers or non-smokers.

Regarding self-reported diet, participants were stratified into those who reported following or not following some dietary advice given by a nutritionist. As for alcohol consumption, self-reported intake was considered regardless of quality and quantity.

### Ethical aspects

This research was approved by the Ethics Committee for Studies on Humans, HC/UFPE (CNPJ: 15.126.437/0016-20), according to the Resolution nº 466/2012 of the National Health Council/Ministry of Health under CAAE: 34950620.7.0000.8807. All study participants signed the informed consent.

### Statistical analysis

Data processing was performed by the software Statistical Package for the Social Sciences (SPSS), version 15.0, for Windows (SPSS Inc., Chicago, IL, USA). Exposure variables were treated as categorical and the outcome variables as continuous.

The Mann Whitney test was used to verify the median differences of LAP and VAI between the groups and, in this analysis, the variables WC and TG were excluded; BMI, WC, TG and HDL-c are part of the LAP and VAI calculation, respectively. After analyzing the difference of medians, the variables were transformed into logarithmic functions to conduct a multivariate linear regression. A level of statistical significance of p ≤ 0.05 was considered. The absence of collinearity between the variables was confirmed by Pearson’s correlation test.

The predictive effect of independent variables on outcomes was assessed by multivariate linear regression analysis using a hierarchic block of variable input. The first block was formed by sociodemographic variables, the second by anthropometric data, the third by the lipid profile, the fourth by variables of the glucose profile and fifth by lifestyle data. All the variables that in the bivariate analysis that presented a p < 0.20 were selected using the enter method.

The block modeling process was used, and the variables that presented p < 0.10 in each block were kept. With LAP as the outcome, the following blocks were formed: 1st Block – sex, self-reported color; 2nd Block – BMI, WHR, WHA; 3rd Block – TC, LDL-c, HDL-c; 4th Block – fasting and postprandial glycemia and HbA1c; and 5th Block – diet and physical exercise.

With VAI, the Blocks were 1st Block – Sex, age, self-reported color; 2nd Block – WHR, WHA; 3rd Block – CT, LDL-c; 4th Block – fasting glucose and HbA1c; and 5th Block – alcohol consumption.

## RESULTS

The population of the present study comprised 1,273 individuals aged between 44 and 92 years, mainly women and old people. Table 1 shows demographic, lifestyle, anthropometric, and biochemical characteristics. Regarding nutritional status and biochemical alterations, most of the analyzed sample was overweight (66.7%) and at risk by WC (82.2%) and WHR (92.5%), LDL-c (62, 6%) and TC (78.9%) were high, and HbA1C (82.9%) and HDL-c (74.1%) were normal.

**Table 1:** Demographic, lifestyle, anthropometric and biochemical characteristics of patients with hypertension

Variables		N	%	CI95%
		1273	100	
Age				
	<60 years	517	40,6	37,9-43,4
	≥60 years	756	59,4	56,6-62,1
Self-reported color				
	White	863	67,8	65,1-70,3
	Non-white	410	32,2	29,7-34,9
Alcohol consumption				
	No	1126	88,5	86,5-90,1
	Yes	147	11,5	9,9-13,5
Smoking				
	No	1135	89,2	87,3-90,8
	Yes	138	10,8	9,2-12,7
Self-reported diet				
	No	853	67,0	64,3-69,6
	Yes	420	33,0	30,4-35,7
Practice of physical exercise				
	No	369	29,0	26,5-31,6
	Yes	904	71,0	68,4-73,5
BMI				
	Not overweight	424	33,3	30,7-36,0
	Overweight	849	66,7	64,0-69,3
WC (risk)				
	No	226	17,8	15,7-20,0
	Yes	1047	82,2	80,0-84,3
WHR (risk)				
	No	96	7,5	6,2-9,2
	Yes	1177	92,5	90,8-93,8
WHA (risk)				
	No	71	25,6	4,4-7,0
	Yes	1202	74,4	93,0 – 95,6
Total cholesterol				
	Normal	268	21,1	18,9-23,4
	High	1005	78,9	76,6-81,1
LDL-c				
	Normal	476	37,4	34,7-40,1
	High	797	62,6	59,9-65,3
HDL-c				
	Normal	943	74,1	71,6-76,4
	Reduced	330	25,9	23,6-28,4
Fasting glucose				
	Normal	625	49,1	46,3-51,9
	High	648	50,9	48,1-53,7
Postprandial glucose				
	Normal	1256	98,7	97,8-99,2
	High	17	1,3	0,8-2,2
HbA1c				
	Normal	1055	82,9	80,7-84,9
	High	218	17,1	15,1-19,3
TG				
	Normal	664	52,2	49,4-54,9
	High	609	47,8	45,1-50,6

BMI: body mass index; CI95%: 95% confidence interval; HbA1c: glycated hemoglobin; HDL-c: high-density lipoprotein-associated cholesterol; LDL-c: low-density lipoprotein-associated cholesterol; TG: triglycerides; WC: waist circumference; WHA: waist-to-height ratio; WHR: waist-to-hip ratio.

Women had higher medians of VAI than men ( Table 2 ). With regard to lifestyle variables, people who reported following a diet had a higher median LAP value. Self-reported non-white individuals with a higher cardiometabolic risk (according to BMI, WHR, WHA, and biochemical parameters) had higher median values of VAI and LAP.

**Table 2:** Association between demographic, lifestyle, anthropometric and biochemical variables with the LAP and VAI

Variables		LAP			VAI		
		Median	IQR	p-value	Median	IQR	p-value
Sex							
	Men	52,5	33,9-74,1	0,062	1,9	1,3-2,7	<0,001
	Women	55,3	38,0-77,3		2,5	1,7-3,4	
Age							
	<60 years	54,9	35,4-83,5	0,506	2,2	1,5-3,2	0,085
	≥60 years	54,6	37,9-73,8		2,4	1,7-3,2	
Self-reported color							
	White	48,5	32,4-68,6	<0,001	2,3	1,6-3,2	0,035
	Non-white	69,2	48,1-92,9		2,4	1,7-3,3	
Alcohol consumption							
	No	54,6	37,3-76,8	0,533	2,4	1,6-3,2	0,005
	Yes	54,6	32,2-78,7		2,0	1,4-2,8	
Smoking							
	No	54,6	37,6-76,8	0,591	2,3	1,6-3,2	0,219
	Yes	57,4	36,4-78,9		2,4	1,7-3,5	
Self-reported diet							
	No	52,6	35,3-73,5	0,001	2,3	1,6-3,2	0,620
	Yes	57,4	40,7-81,8		2,3	1,6-3,2	
Practice of physical exercise							
	No	54,2	35,4-71,9	0,216	2,3	1,6-3,2	0,665
	Yes	54,9	37,1-78,7		2,3	1,6-3,2	
BMI							
	Not overweight	35,7	24,3-52,1	<0,001		–	
	Overweight	64,4	46,4-87,0					
WHR (risk)							
	No	30,6	20,1-46,6	<0,001	1,8	1,2-2,5	<0,001
	Yes	56,4	39,2-78,7		2,4	1,6-3,3	
WHA (risk)							
	No	19,2	12,6-23,6	<0,001	1,4	1,0-1,9	<0,001
	Yes	56,2	39,8-78,4		2,4	1,7-3,3	
Total cholesterol							
	Normal	41,0	26,8-49,4	<0,001	1,7	1,2-2,6	<0,001
	High	57,8	40,8-80,7		2,5	1,7-3,4	
LDL-c							
	Normal	48,9	31,2-73,6	<0,001	2,0	1,3-3,0	<0,001
	High	56,9	40,0-8,2		2,5	1,8-3,3	
HDL-c							
	Normal	51,8	34,2-73,8	<0,001		–	
	Reduced	60,3	43,2-85,9				
Fasting glucose							
	Normal	48,8	31,8-70,4	<0,001	2,2	1,5-3,1	<0,001
	High	58,7	42,7-83,0		2,4	1,7-3,4	
Postprandial glucose							
	Normal	54,6	36,6-76,8	0,097	2,3	1,6-3,2	0,298
	High	67,0	48,7-109,8		2,7	1,6-2,7	
HbA1c							
	Normal	53,1	35,7-73,6	<0,001	2,3	1,6-3,2	0,004
	High	61,8	43,8-90,0		2,5	1,7-3,7	

BMI: body mass index; IQR: interquartile range; HbA1c: glycated hemoglobin; HDL-c: high-density lipoprotein-associated cholesterol; LDL-c: low-density lipoprotein-associated cholesterol; TG: triglycerides; WC: waist circumference; WHA: waist-to-height ratio; WHR: waist-to-hip ratio.

Statistical significance according to Mann-Whitney test (p ≤ 0.05).

In the linear regression model ( Table 3 ), being non-white, overweight, at risk by WHA, and WHR and high values of TC, LDL-c, fasting glucose, and HbA1c increased the values of LAP, while serum levels considered normal of HDL-c lowered this index. Regarding VAI ( Table 4 ), in all models there was an increase in its values. The regression models used explained 30.7% and 10.5% of the changes in LAP and VAI, respectively.

**Table 3:** Multiple linear regression analysis of variables associated with LAP

Variables		LAP					
		β a unadjusted	p-value	β adjusted	[CI95%]	p-value	R² (%) b
Model 1							0,4 (10,4)
	Self-reported color (non-white)	23,6	<0,001	23,6	[19,2-27,9]	<0,001	
Model 2							
	BMI (Overweight)	30,5	<0,001	20,1	[16,7-23,1]	<0,001	13,0 (23,4)
	WHR (with risk)	26,8	<0,001	14,5	[9,1-19,6]	<0,001	
	WHA (with risk)	41,6	<0,001	21,7	[16,6-25,8]	<0,001	
Model 3							4,6 (28,0)
	Total cholesterol (high)	17,6	<0,001	20,3	[15,0-25,6]	<0,001	
	LDL-c (high)	5,7	<0,001	8,0	[13,1-2,9]	<0,001	
	HDL-c (normal)	-9,2	<0,001	-8,9	[-12,5-5,1]	<0,001	
Model 4							2,3 (30,3)
	Fasting glucose (high)	12,3	<0,001	6,5	[3,3-9,8]	<0,001	
	Postprandial glucose (high)	26,2	0,097	13,9	[-13,3-42,8]	0,056	
	HbA1c (high)	12,6	<0,001	6,0	[1,1-11,5]	0,012	
Model 5							0,4 (30,7)
	Self-reported diet (yes)	7,0	0,001	3,7	[-0,4-7,2]	0,044	

a Non-standardized regression coefficient.

b Determination coefficient.

BMI: body mass index; HbA1c: glycated hemoglobin; HDL-c: high-density lipoprotein-associated cholesterol; LDL-c: low-density lipoprotein-associated cholesterol; WHA: waist-to-height ratio; WHR: waist-to-hip ratio.

Model 1: adjusted by sex; model 5: adjusted by practice of regular physical exercise reported.

Reference categories for categorical variables: self-reported color (white); BMI (not overweight); WHR (without risk); WHA (without risk); total cholesterol (normal); LDL-c (normal); HDL-c (reduced); fasting glucose (normal); postprandial glucose (normal); HbA1c (normal); self-reported diet (no).

**Table 4:** Multiple linear regression analysis of variables associated with VAI

Variables		VAI					
		β a unadjusted	p-value	β adjusted	[CI95%]	p-value	R² (%) b
Model 1							3,1 (3,1)
	Sex (women)	0,5	<0,001	0,5	[0,4-0,7]	<0,001	
	self-reported color (non-white)	0,2	0,035	0,2	[0,0-0,4]	0,048	
Model 2							3,0 (6,1)
	WHR (with risk)	0,6	<0,001	0,6	[0,4-0,8]	<0,001	
	WHA (with risk)	0,9	<0,001	0,7	[0,4-1,0]	<0,001	
Model 3							3,1 (9,2)
	Total cholesterol (high)	0,7	<0,001	0,6	[0,4-0,9]	<0,001	
Model 4							1,3 (10,5)
	Fasting glucose (high)	0,3	<0,001	0,2	[0,0-0,3]	0,043	
	HbA1c (high)	0,4	<0,004	0,3	[0,0-0,5]	0,011	

a Non-standardized regression coefficient.

b Determination coefficient.

HbA1c: glycated hemoglobin; WHA: waist-to-height ratio; WHR: waist-to-hip ratio.

Model 1: adjusted by age; model 3: adjusted by LDL-c; model 4: adjusted by alcohol consumption.

Reference categories for categorical variables: sex (men); self-reported color (non-white); WHR (without risk); WHA (without risk); total cholesterol (normal); fasting glucose (normal); HbA1c (normal).

## DISCUSSION

The present analysis found, in agreement with previous studies carried out with men and women aged between 25 and 65 years from Indonesia ( 22 ) and individuals aged between 18 and 90 years treated in primary health care ( 23 ) that higher medians of LAP and VAI are associated with changes in anthropometric and biochemical parameters, although the population studied in previous analyses was not composed of individuals with hypertension. Also, when inserted into the linear regression model, the anthropometric and changed biochemical variables in the present study, except for postprandial glucose in the LAP model, significantly increased the two indices used.

It is known that the change in the lifestyle of populations changed the body composition of individuals, resulting mainly in an increase in body fat, especially in the abdominal region. Also, visceral adipose tissue, as it is considered a metabolically active component, secretes pro-inflammatory adipokines that increase the risk for cardiovascular and metabolic disorders ( 24 , 25 ).

LAP is proposed as an index that reflects the physiological and anatomical changes that are associated with visceral fat deposition ( 26 ), with a predictive power superior to other parameters, such as BMI, to identify risk for cardiovascular outcomes and diabetes ( 22 , 23 ), as well as all-cause mortality prediction ( 27 , 28 ). In previous national studies ( 8 , 23 , 29 ), LAP was found to be significantly associated with classical cardiovascular biomarkers, which is in agreement with our findings.

The VAI had a significant correlation with visceral adiposity, showing superiority in relation to the components that enter its equation in terms of discrimination of cardiovascular and cerebrovascular events in a previous study ( 9 ). Furthermore, it has been suggested that such an index is a simple tool for the assessment of adipose tissue dysfunction ( 30 ). In order to support the use of the VAI as an additional risk indicator of cardiovascular outcomes, a long-term prospective study found that this index was independently associated with a high ten-year CVD risk, particularly in men without previous CVDs ( 31 ).

Unlike the indices discussed here, excess weight assessed by BMI is not a good indicator of body adiposity distribution. However, it may indicate a higher risk for CVD as it is associated with cardiometabolic alterations ( 32 ). As 66.7% of individuals were overweight, this high prevalence can be explained by the fact that high body weight is involved in the etiology of AH through multiple physiological mechanisms that lead to endothelial dysfunction present in the hypertensive disease ( 33 ).

It is known that excess adiposity is involved with changes in biochemical markers, exacerbating the risk of atherosclerosis ( 6 ). The results obtained in this analysis emphasize LAP and VAI as predictors of cardiometabolic risk by showing a directly proportional relation to altered laboratory markers and, in the case of LAP, inversely with HDL-c, which plays an important role in reverse transporting of cholesterol and several other beneficial biological properties, which in turn enhance its protective effect against CVD ( 34 ). In the present study, only the altered postprandial blood glucose in the LAP model did not significantly increase the index, but it is important to note that only 1.3% of hypertensive individuals had a high value for this parameter, which may have caused the not significant results.

When evaluating the difference in indices by sex, women had a higher median VAI. It is known that sex steroids play a role in both the distribution and function of adipose tissue ( 35 ). Furthermore, after menopause, there is a reduction in estrogen levels, increased adiposity and inflammatory markers that can exacerbate metabolic risk ( 22 ). As most of the present sample consisted of elderly women, it is assumed that they would be in the post-menopausal period and, therefore, susceptible to the aforementioned hormonal and physical changes.

The present study identified that individuals that self-declared as non-white had higher means of adiposity indices. A strong relationship between the worst socioeconomic level and the black or mixed race ( 36 ) was previously established in the literature, which may imply less access to information and services related to health, which may in turn lead to deleterious changes ( 37 ), such as the one identified here.

Another finding was that individuals who reported following dietary guidelines had increased LAP values and that it was associated with an increase in the index in the regression model, while individuals self-reported as non-alcoholic had a higher median value of VAI. It is important to highlight the fragility of these data, since these are information referred to in an outpatient care context to which individuals may have distorted the response because they were being approached by a health professional. In addition, the cross-sectional design of the study precludes causal associations. Furthermore, an association between excessive consumption of alcoholic beverages and the risk of CVD was previously established in the literature ( 38 ).

This study has as a limitation the fact that VAI and LAP were not developed for the Brazilian population. However, it is expected that the results found may stimulate the development of clinical trials and prospective cohorts to support the definition of cutoff points for adiposity indices to identify cardiometabolic risk in Brazilians. Also, as the study was conducted only with individuals diagnosed with hypertension, caution is suggested in extrapolating the results to the population without hypertension. Despite this, the present analysis encompassed a large number of individuals and assessed cardiometabolic risk using simple indicators that are applicable in clinical care.

The results of this study show that LAP and VAI are associated with anthropometric and biochemical markers of cardiometabolic risk and that they increased both visceral adiposity indices, indicating that individuals predisposed to greater risk for adverse outcomes can be identified by them.
